# MindHike, a Digital Coaching Application to Promote Self-Control: Rationale, Content, and Study Protocol

**DOI:** 10.3389/fpsyt.2020.575101

**Published:** 2020-10-09

**Authors:** Mathias Allemand, Lara Keller, Benjamin Gmür, Victoria Gehriger, Timon Oberholzer, Mirjam Stieger

**Affiliations:** ^1^Department of Psychology and University Research Priority Program Dynamics of Healthy Aging, University of Zurich, Zurich, Switzerland; ^2^Wia Ventures GmbH, Gränichen, Switzerland; ^3^Department of Psychology, Brandeis University, Waltham, MA, United States

**Keywords:** digital coaching, promotion of self-control, process model of self-control, conscientiousness, smartphone application (App), MindHike

## Abstract

This protocol describes a study that will test the effectiveness of a 7-week non-clinical digital coaching intervention to promote self-control. The goal of the coaching is to support and guide people who are willing and motivated to improve their self-control with the help of the smartphone application MindHike. The coaching is based on a process model of self-control and aims to target five groups of self-control strategies. The goal of the study is to examine the effectiveness of the digital coaching intervention. A single-arm study design with pre-test, post-test and 2-month follow-up assessments and process assessments will be used to evaluate the 7-week digital coaching intervention. The digital coaching includes 49 daily lessons that are organized along 7 weekly core themes. Study participants will be at least 150 adults aged 18 years and older who are willing and motivated to improve their self-control using the MindHike application. This is the first study testing the effectiveness of a digital coaching intervention to promote self-control. Given that this approach proves effective, it could be easily implemented in various non-clinical settings such as education, health, relationship, and work, and in clinical settings. Due to its digital low-threshold character, it could also reach large numbers of people.

## Introduction

Self-control is the ability to suppress or control impulsive actions, emotions, and desires in favor of desired alternatives. It enables people to reach concurrent or long-term goals (e.g., like sticking to a diet or passing difficult exams) despite the presence of short-term desires and distractions (1–3). The term self-control is broadly used both by researchers and lay persons (4) and there exists a broad range of definitions, concepts, and measures of self-control [see e.g., (5, 6) for reviews]. Numerous research findings suggest that high self-control is related to various positive outcomes including better academic success and work performance, better interpersonal functioning, greater well-being and adjustment, better health outcomes and longevity, and reduced economic costs (7–11) [see de Ridder et al. (12) for a meta-analytic review]. In contrast, low self-control was linked to various problematic behaviors and unhealthy actions such as overeating, substance abuse, criminality, and impulsive buying (13).

Research findings also indicate that self-control is not fixed and unchangeable in adulthood, but can evolve over the lifespan (14). Initial evidence even suggests that changes in self-control through the adolescent years are associated with positive social relationships and success at work years later in life (15). Hence, it is not surprising that efforts have been made to design interventions and trainings to promote self-control in adolescence and adulthood through learning, practice, and effort (16). Experimental and intervention research suggests that regular practice of self-control may improve self-control with small-to-medium effects (17–19). Interventions are a promising avenue for promoting self-control and may help to contribute to various positive outcomes. This protocol describes the rationale and content of a digital coaching application to promote self-control as well as a study to examine the effectiveness of this app-based coaching. The coaching intervention is strongly based on the process model of self-control (5).

### The Process Model of Self-Control

The process model of self-control (5) posits that impulses develop in an iterative cycle and tend to grow stronger over time. The cycle starts with a specific situation, moves on to attentional deployment, followed by appraisal of the situation, and ends with a response tendency. This model organizes self-control strategies in five groups by considering the timeline of the developing tempting impulse. At the earliest stage of the impulse generation, there are two situational strategies that describe how people can select and modify circumstances to facilitate actions that help them to achieve their long-term goals: (a) Situation-selection strategies entail the deliberate choice of situations that favor goal-oriented valuation systems over temptation-oriented valuation systems. (b) Situation-modification strategies can be used if situation selection is not possible. These strategies refer to the intentional modification of situations to facilitate goal achievement.

At later stages of the impulse generation, there are three intrapsychic strategies that describe how people can use attention, cognitive change, or response modulation to get closer to their goal. When situation selection or modification is not possible, (c) attentional deployment can be used to focus on features of the situation that facilitate self-control. When confrontation with temptations is unavoidable, (d) cognitive-change strategies can be used to diminish undesired impulses and amplify desired ones. Cognitive change involves thinking differently about the situation. At the final stage of impulse generation, (e) response modulation strategies refer to intentionally suppress undesirable impulses or amplify desirable ones [see Duckworth et al. (5) for more details].

This process model served as a conceptual guideline for the development of the coaching intervention components to promote self-control. The components target each stage in the process model of self-control an include both situational strategies as well as intrapsychic strategies. Furthermore, the process model may serve as a useful intervention model that may be easy to understand for coaches and coachees in applied settings.

### Digital Coaching to Promote Self-Control

There exist different approaches to promote and strengthen self-control ranging from broad to more specific approaches [e.g., (16)]. For instance, a recently proposed intervention framework suggests considering four common change factors as useful heuristic principles when designing personality change interventions (20). First, intervention efforts should actuate discrepancy awareness which refers to the idea that desired changes can be most effectively targeted when people realize that there is a gap between who they are and how they want to be, and what they need to change. Second, the intervention should activate strengths and resources which initiate and maintain positive feedback circuits and expectations. Third, interventions should target and increase one's awareness of beliefs, expectations, and motives in order to realize insight by learning how to systematically reflect thoughts, feelings, and behaviors. Fourth, the intervention should teach how to practice new behaviors.

Another recent article describes several ways to promote self-control in children and adolescents including simple strategies that help to create habits and reduce conflicts with competing temptations (21). Three different methods are distinguished: (a) practicing self-control (e.g., engaging in actions that require effort to sustain or that are counter-habit), (b) goal attainment (e.g., implementation intentions, mental contrasting), and (c) mental transformation (e.g., transforming “hot” temptations to “cold,” shifting construal level). Also, a recent review discusses cognitive and behavioral training interventions to promote self-control (19). The discussed self-control training interventions included effort exposure, reward discrimination, reward bundling, interval schedules of reinforcement, impulse control training, and mindfulness training.

Although meta-analytic work suggests that different approaches can improve self-control (17, 18), evidence-based coaching interventions that support and guide people in their daily life to improve and strengthen their self-control are lacking. Coaching refers to “unlocking people's potential to maximize their own performance. It is helping them to learn rather than teaching them” [(22), pp. 12–13]. Typically, coaching interventions are delivered in-person by “real” coaches or interventionists. Advances in wireless devices and mobile technology offer many opportunities for delivering interventions without engagement of real interventionists and coaches. Smartphone applications are particularly attractive and innovative ways for delivering coaching interventions, providing support, and guiding people in a timely, adaptive, and ecologically attuned manner in daily life (23). This work focuses on coaching people in a digital way using a smartphone application. Especially in the area of self-assessment, self-monitoring, and self-change efforts, numerous smartphone applications can be found in the App Store or Google Play Store, also apps with the goal to promote self-control. However, almost all applications do not meet scientific criteria and their effectiveness has not been systematically tested.

### Aims and Hypotheses

The first aim of the study is to examine the effectiveness of the digital coaching intervention to promote self-control. The focus of this initial research is on the within-person evaluation of the effectiveness of the digital coaching and not on the between-person comparison with active or passive control groups. As a first step in the evaluation process, this study will focus on a non-clinical sample of people without mental health conditions. The first outcome hypothesis is that participation in the digital coaching would lead to increases in self-control. The second outcome hypothesis is that participation in the digital coaching would also lead to increases in conscientiousness. The third outcome hypothesis is that participation in the digital coaching would lead to increases in satisfaction with life in general and satisfaction with health, work, and relationships, depending on the selected target area of the coaching (i.e., health, work, relationships, other) and the individual intervention goal.

The second aim is to examine underlying within-person processes and mechanisms that improve the outcomes of the digital coaching to promote self-control. The first process hypothesis is that the amount of accomplished tasks is positively related to increases in self-control. The second process hypothesis is that the difficulty of tasks is negatively related to increases in self-control. The third process hypothesis is that the amount of learning is positively related to increases in self-control. The fourth process hypothesis is that increases in self-control are related to increases in conscientiousness.

## Methods and Analysis

### Study Design

A single-arm study design with repeated measures will be used to evaluate the 7-week digital coaching to promote self-control (49 days). Single-arm trials, in which all participants are enrolled in the same treatment, are common in the first phases of the evaluation process of a treatment and are characterized by an exploratory nature. The current study is not designed to obtain definitive conclusions about the effectiveness of the digital intervention but rather to provide initial evidence. Outcome assessments will be completed at pre-test, at the end of the coaching (~8 weeks later), and at the 2-month follow-up after the coaching (~16 weeks after start of the study). Moreover, intensive longitudinal assessments will include end-of-week and daily assessments during the 7-week intervention phase. The design and procedure of the study is shown in Table 1.

**Table 1:** Design and procedure of the study.

**Pre-test (T1)**	**Intervention**	**Post-test (T2)**	**Follow-up (T3)**
**Day 0 (week 1)**	**Day 1–49 (week 1–7)**	**Day 50 (week 8)**	**Day 106 (week 16)**
*Self-reports*	*Daily coaching session*	*Self-reports*	*Self-reports*
–Primary outcome assessment	–At individually preferred time	–Primary outcome assessment	–Primary outcome assessment
–Secondary outcome assessment	*Self-reports* –Process assessment	–Secondary outcome assessment	–Secondary outcome assessment
*Observer reports*		*Observer reports*	*Observer reports*
–Outcome assessment		–Outcome assessment	–Outcome assessment

*The pre-test (T1) will also include the informed consent and the screening assessment*.

### Participants and Recruitment Strategy

The targeted sample will include at least 150 adults who will install the MindHike app on their smartphones, give informed consent, pass the screening, complete the pre-test assessment, start with the digital coaching program, and complete the post-test and follow-up assessments. Participants will complete an online screening that checks for the eligibility criteria. The inclusion criteria of the study are: (a) aged 18 years or older; (b) owner of a smartphone (Android or iOS) with mobile internet connection; and (c) interested and motivated to participate in a digital coaching to promote and strengthen their self-control ability. The exclusion criteria of the study are: (a) being in psychotherapeutic or psychiatric treatment; and/or (b) having mental health problems (i.e., depression). Excluded candidates with mental health problems will be provided with an information and contact details of psychological counseling services.

We will primarily use university mailings, social media, and word-of-mouth advertising to recruit study participants. Interested people will be directed to the website of the project (https://mindhike.ch) to receive detailed information about the study aims, the intervention, the eligibility criteria, the assessments, reimbursement, and data protection. Individuals who are interested in participating in the study will contact the study team via e-mail and will receive a link to an online survey to complete the informed consent, the screening, and pre-test assessments. Eligible participants will also receive information on how to download the MindHike application.

### Procedure

The procedure of the study is shown in Table 1. After giving informed consent and passing the screening assessment, participants will be directed to the pre-test assessment. After having completed the pre-test assessment, participants will download and install the mobile application and will start with the first app-based coaching session. The duration of the intervention is 7 weeks. At every last day of each week, participants will be asked to complete a short self-control measure. After the intervention, participants will be asked to complete the post-test assessment and then after 2 months, participants will be contacted to complete the follow-up assessment (Table 1). To reduce the risk of missing data, an online survey platform (https://www.unipark.com/) external to the MindHike application will be used to obtain informed consent and to assess the outcome variables. Online survey platforms provide “force response” options which require the respondents to answer questions before they can continue the survey. The weekly process assessments will be completed using the MindHike app.

### The MindHike Application

**Figure 1:**
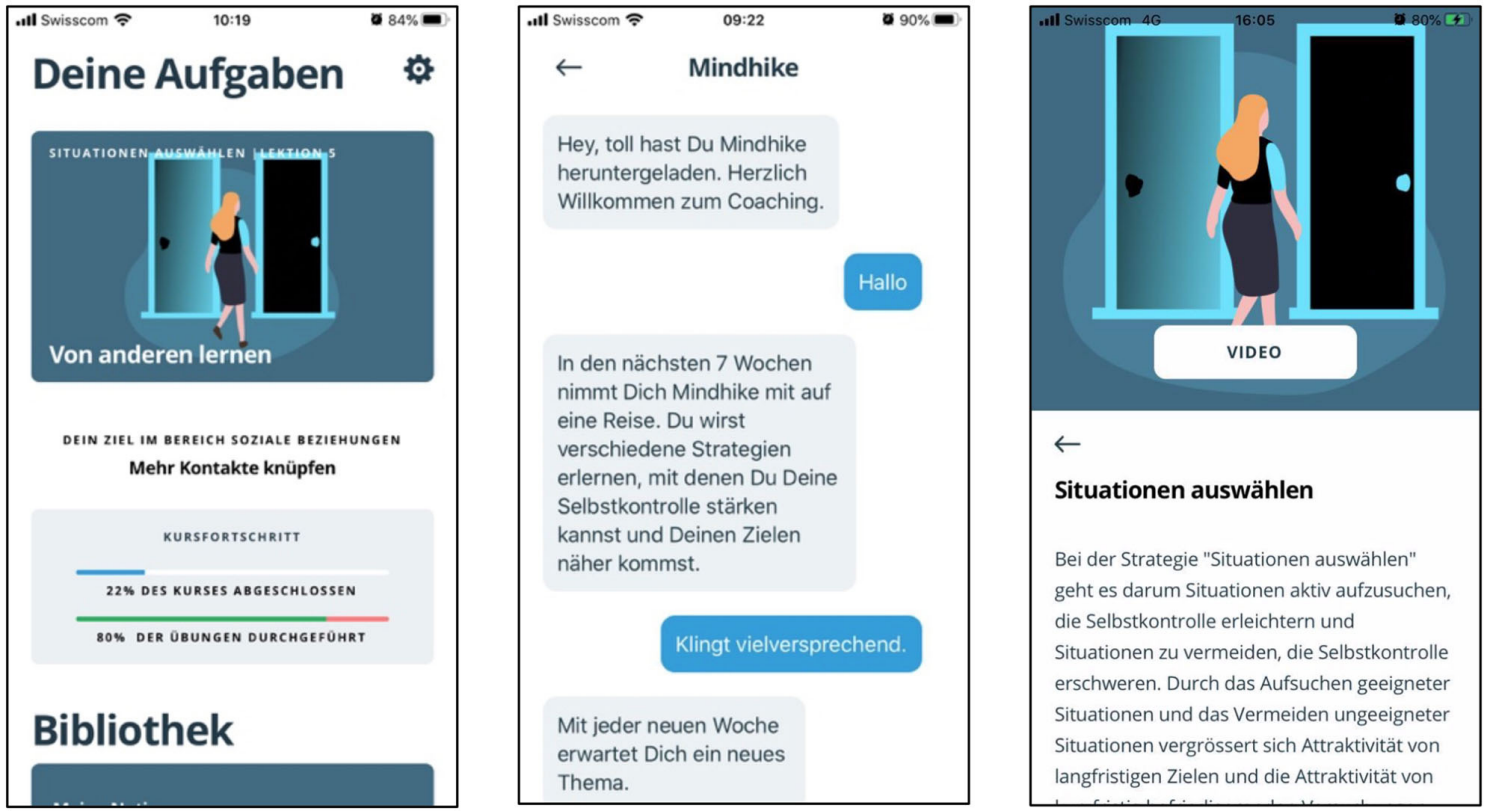
The MindHike application (in German) with the dashboard including access to a daily session, coaching goal, course progress, and access to the media library **(left)**, short dialogue from the first daily coaching session **(middle)**, element from the media library **(right)**.

The MindHike app was developed to serve as a digital coach in order to guide and support individuals in their self-imposed goal to increase their self-control. The entire coaching is delivered via the MindHike app, although the app could also be combined with a real face-to-face coaching. The coaching implementation procedure is as follows: Participants will be contacted by the MindHike app every day for 7 weeks. The daily timing of the coaching can be customized by each participant. At the preselected time, participants will receive a reminder to start the coaching session. The daily coaching sessions consist of short interactions of a few minutes with the digital coach. Each session comprises of a short dialogue between the digital coach and the participant in which the digital coach provides educational components, microinterventions, and reminders to practice the tasks (Figure 1). Microinterventions are small psychological interventions including specific tools and techniques to help people modify and change their typical behaviors in their everyday contexts and help them to initiate and maintain the change process (24). The digital coach also praises participants' progress to motivate them and support the change process. The content of MindHike is structured as follows: 49 daily lessons are organized along 7 weekly core themes. These core themes consist of an introductory week to introduce the digital coaching, followed by five groups of self-control strategies, and a closing week (Table 2). The MindHike app includes three types of lessons: (a) education (short film clips / animations to introduce the process model of self-control and the five groups of self-control strategies), (b) tasks (2–3 microinterventions per weekly core theme), and (c) practice (reminders to practice the introduced tasks). For each of the five groups of self-control strategies, we selected evidence-based microinterventions that can be implemented using short dialogues (Table 3). The Mindhike app was developed based on the react native framework (https://www.reactnative.dev). This allows to publish the application on Android and on iOS. The user input data is stored on the smartphone as well as on a server. All study participants receive an individual login code at the beginning of the study. This code is used as unique ID with which the collected data can be matched.

**Table 2:** Schedule of the weekly core themes and daily lessons of MindHike.

**Day**	**Core theme**	**Lesson**	**Type**	**Title**
1	Introduction	1	Education	*A short introduction*
2	Introduction	2	Task	Setting the goal
3	Introduction	3	Practice	Setting the goal—in action
4	Introduction	4	Task	Designing a positive future
5	Introduction	5	Practice	Designing a positive future—in action
6	Introduction	6	Task	Recognizing your own strengths
7	Introduction	7	Practice	Recognizing your own strengths—in action
8	Situation selection	1	Education	*Situation selection*
9	Situation selection	2	Task	Observing yourself
10	Situation selection	3	Practice	Observing yourself—in action
11	Situation selection	4	Practice	Observing yourself—in action
12	Situation selection	5	Task	Learning from others
13	Situation selection	6	Practice	Learning from others—in action
14	Situation selection	7	Practice	Learning from others—in action
15	Situation modification	1	Education	*Situation modification*
16	Situation modification	2	Task	Optimization through adding
17	Situation modification	3	Practice	Optimization through adding—in action
18	Situation modification	4	Practice	Optimization through adding—in action
19	Situation modification	5	Task	Optimization through removal
20	Situation modification	6	Practice	Optimization through removal—in action
21	Situation modification	7	Practice	Optimization through removal—in action
22	Attentional deployment	1	Education	*Attentional deployment*
23	Attentional deployment	2	Task	If-then plans
24	Attentional deployment	3	Practice	If-then plans—in action
25	Attentional deployment	4	Practice	If-then plans—in action
26	Attentional deployment	5	Task	Breathing space
27	Attentional deployment	6	Practice	Breathing space—in action
28	Attentional deployment	7	Practice	Breathing space—in action
29	Cognitive change	1	Education	*Cognitive change*
30	Cognitive change	2	Task	Take a step back in your thoughts
31	Cognitive change	3	Practice	Take a step back in your thoughts—in action
32	Cognitive change	4	Practice	Take a step back in your thoughts—in action
33	Cognitive change	5	Task	Change through acceptance
34	Cognitive change	6	Practice	Change through acceptance—in action
35	Cognitive change	7	Practice	Change through acceptance—in action
36	Response modulation	1	Education	*Response modulation*
37	Response modulation	2	Task	Break habits
38	Response modulation	3	Practice	Break habits—in action
39	Response modulation	4	Practice	Break habits—in action
40	Response modulation	5	Task	Every beginning is hard
41	Response modulation	6	Practice	Every beginning is hard—in action
42	Response modulation	7	Practice	Every beginning is hard—in action
43	Closing	1	Education	*Use the toolbox*
44	Closing	2	Practice	Situation selection—in action
45	Closing	3	Practice	Situation modification—in action
46	Closing	4	Practice	Attentional deployment—in action
47	Closing	5	Practice	Cognitive change—in action
48	Closing	6	Practice	Response modulation—in action
49	Closing	7	Education	Keep on going!

**Table 3:** Microinterventions of MindHike.

**Core theme**	**Title**	**Brief description**	**References**
Introduction	Setting the goal	Selecting the target area of the digital self-control coaching (health, work, relationships) and setting a goal for the 7 weeks with SMART principles	(25, 26)
Introduction	Designing a positive future	Imagining in vivid detail a positive outcome of attaining the coaching goal and then bringing to mind a negative obstacle that presently stands in the way of behaving self-controlled (mental contrasting)	(27, 28)
Introduction	Recognizing your own strengths	Thinking about one's own individual strengths and resources (e.g., personal skills) that can facilitate self-control (strength-orientation)	(29)
Situation selection	Observing yourself	Watching and thinking about one's own behavior and expression of thought and feeling reflection (self-reflection) in two types of situations: (a) avoidance of situations that can contain triggers and reinforcers of temptations, (b) approach of situations that can reinforce desired impulses	(30–32)
Situation selection	Learning from others	Watching and thinking about others' behavior and expression of thought and feeling (model learning) in two types of situations: (a) avoidance of situations that can contain triggers and reinforcers of temptations, (b) approach of situations that can reinforce desired impulses	(31–33)
Situation modification	Optimization through adding	Changing physical or social circumstances by adding helpful things (objects or people) to the situation or by bundling complementary and not-so-bad temptations with desired behaviors	(34)
Situation modification	Optimization through removal	Changing physical or social circumstances by removing triggers of temptations (objects or people) from sight rather than trying to resist them directly	(35)
Attentional deployment	If-then plans	Learning to direct attention to specific situations by means of implementation intentions that specify the when, where, and how of goal striving in advance	(36–38)
Attentional deployment	Breathing space	Learning to direct attention through regular mindfulness meditation to manage temptation and unwanted impulses (breathing exercise)	(39–41)
Cognitive change	Take a step back in your thoughts	Changing how one thinks about or appraises a given situation by taking a step back and creating (a) unattractive images for temptations and unwanted impulsive actions, or (b) vivid and attractive images for desired impulses and actions (mental training/ reframing)	(42, 43)
Cognitive change	Change through acceptance	Changing how one thinks about or appraises a given situation by learning to accept that different thoughts and feelings may appear	(40, 44)
Response modulation	Break habits	Disrupting behavioral habits related to self-control by replacing routines with new or alternatives activities that should be (a) easy to implement in daily life, (b) require a simple “yes or no” decision and (c) should not be too time-consuming (habit change)	(45, 46)
Response modulation	Every beginning is hard	Activate behavioral activities by writing down five behavioral activities that promotes goal striving, weighting these activities from easy to difficult to implement, and then start with the simplest one (behavioral activation)	(47, 48)

### Screening Assessment

The screening assessment will test for the inclusion criteria: (a) aged 18 years or older; (b) owner of a smartphone (Android or iOS) with mobile internet connection; and (c) interested and motivated to participate in a digital coaching to promote and strengthen self-control.

Moreover, we will screen for the two exclusion criteria: Participants will be asked whether they currently are in psychotherapeutic or psychiatric treatment. Also, the Depression Scale [ADS-K; (49)] will be used to assess mental health problems. Individuals with scores above the cut-off value in the ADS-K (≥19) will be excluded and are provided with information and contact details of the psychological counseling service of the University of Zurich for appropriate treatments and they can also contact the study office for more information.

### Primary Outcome Self-Report Assessment

The Self-Control Scale [SCS; (10)] will be used to measure individual differences in the ability to control impulses. The self-discipline facet scale from the International Personality Item Pool [IPIP; (50, 51)] will be used. The conscientiousness scale from the Big Five Inventory-2 [BFI-2; (52)] will be used to measure individual differences in conscientiousness. These self-report measures will be used at pre-test, post-test and follow-up assessment.

### Secondary Outcome Self-Report Assessment

The other Big Five traits will be measured using the BFI-2 (52) to examine potential transfer effects of the digital coaching on personality traits other than conscientiousness. Satisfaction with life in general will be assessed using the Satisfaction with Life Scale [SWLS; (53)] and single-item measures to assess satisfaction with health, work, and relationships. Finally, the Rosenberg Self-Esteem Scale [RSES; (54)] will be used to assess individual differences in self-esteem.

### Outcome Observer Reports Assessment

In addition to self-reports, observer reports by close others will be used. At the beginning of the study, participants will be asked to share a link to the online observer report questionnaires with at least three close friends, family members, or their intimate partner. Observer reports include the SCS (10) and a short form of the BFI-2 [BFI-2-S; (55)], type and closeness of the relationship and time spent with the target person. A short version of the Behavioral Indicators of Conscientiousness [BIC; (56)] will be used to rate observable conscientious behaviors. Observer reports will be assessed at pre-test, post-test, and follow-up assessment.

### Process Self-Report Assessment

During the 7-week intervention, there will be weekly assessments of the SCS (10) every last day of the week. The instruction will be: “For each statement, indicate how much it applied to you in the past week.” Additionally, 1 day after each practice session, participants will be asked (a) whether they completed the task (yes, no); (b) if not, why they could not complete the task (no time, task not understood, no desire, other reasons); (c) whether they could learn anything from the task (ranging from “little” to “a lot”) and (d) how difficult the task was for them (ranging from “simple” to “difficult”).

### Statistical Analysis

Missing data analysis will be performed and the type of missing data (i.e., random or non-random) will guide the handling of missing data (e.g., data imputation, estimation techniques). Longitudinal multilevel modeling and longitudinal structural equation modeling will be used to analyze the longitudinal, nested data structure and change over time (57–59). Both data analytic methods are specifically suitable to model change explicitly as a function of time and can be used to formulate equivalent models, providing identical estimates for the collected data. Both methods include estimation procedures in which imputation of missing data is not necessary. Separate models will be analyzed including the outcome assessments at pre-, post- and follow-up (self-reports and observer reports), and the outcome self-report assessments. Predictor/control variables will be added to the models to examine how individual growth will be moderated by variables such as demographic variables. The statistical modeling programs Mplus (60), and updated R packages (R Core Team, Vienna Austria) will be used to estimate the growth curve models.

### Power Analysis

To assure adequate power for the primary outcome hypotheses, we calculated the sample size for a traditional ANOVA with repeated measures, within factors using the G^*^Power program (61). Assuming an α error level of 0.05, a statistical power (1-β) of 0.80, three assessments, and a correlation of 0.40 between the assessments, a sample size of at least 136 participants is required to detect a small effect (Cohen's *d* = 0.24). Computing power for longitudinal multilevel analyses and longitudinal structural equation models, which is the case in this study, is more complex. As such, this power analysis only gives a rough overview of the required sample size. Therefore, the sample will include at least 150 participants. We will over recruit participants (+25%) to be prepared for potential dropouts over time. Should drop-out rates be higher than expected, we may recruit additional participants to ensure sufficient statistical power.

## Discussion

Research has consistently demonstrated the importance of self-control for the prediction of important life outcomes in the domains of health, work, and relationships [e.g., (9)]. Also, individual differences in self-control are seen as a key consideration for policy-makers who seek to enhance the physical and financial health of the population (62, 63). Although available evidence suggests that interventions are a promising avenue for promoting self-control (17, 18), an evidence-based digital coaching intervention that supports and guides people in their daily life to improve and strengthen their self-control is lacking so far. The planned study is the first one testing the effectiveness of a digital coaching intervention that supports and guides people on how to work on and improve their self-control ability. Understanding the short-term changeability of self-control in everyday life and examining whether intended changes in self-control can be maintained (or rather revert over time) is an important research goal and complements previous work on long-term developmental change in self-control across adulthood [e.g., (14)]. This is particularly important as level and change in self-control can have a powerful impact on people's lives (9, 15).

This study will advance our understanding of the underlying processes and mechanisms that improve the outcomes of the digital coaching to promote self-control. Furthermore, the study will contribute to a better understanding of coaching in everyday life by means of a smartphone application. Supporting and coaching individuals to promote their self-control ability with the help of a “pocket coach” is an innovative approach how to provide support to people in their everyday life contexts. As empirical evidence of digital coaching interventions is still sparse, the planned study will advance our understanding of the effectiveness of digital interventions and whether they can serve as useful tools to deliver such interventions. This study will represent an initial effort to evaluate the effects of the digital coaching using a simple study design. Future research is needed to systematically investigate the effectiveness of the intervention using comparative designs such as randomized controlled trials with different control conditions and the comparison with existing self-control interventions (16). Given the intervention approach proves effective, the digital coaching application can not only be used to complement and extend existing face-to-face coaching sessions, but can also provide new means to offer smartphone app-based coaching interventions in a scalable fashion where a face-to-face coaching approach is not feasible due to limited reach, personnel, or budget. The digital coaching intervention with the MindHike app could easily be adapted and used as a preventive coaching tool in various non-clinical settings such as education, health, relationship, and work. Due to its digital low-threshold character, it could also reach large numbers of people. Future research should also examine the effectiveness of the digital intervention in clinical samples. People with mental health conditions such as impulse control disorders or personality disorders tend to show problems with impulse control [e.g., (10, 64)].

## Ethics Statement

The study involving human participants was reviewed and approved by Ethics Committee of the Philosophical Faculty of the University of Zurich, Switzerland (Number of Approval: 19.6.3, Date of Approval: June 5, 2019). The patients/participants provided their written informed consent to participate in this study.

## Author Contributions

LK, BG, VG, and MA designed the psychological part of the MindHike app. TO and WIA Ventures GmbH designed the user experience and the technical part of the MindHike app. MS was responsible for the English translation of the MindHike app. MA and LK designed the study. MA conceptualized and drafted the manuscript. MS provided input on drafts of the manuscript. MA made revisions. All authors read and approved the final manuscript.

## Conflict of Interest

TO was managing director of the start-up company Wia Ventures GmbH, which designed the user experience and the technical part of MindHike. The remaining authors declare that the research was conducted in the absence of any commercial or financial relationships that could be construed as a potential conflict of interest.
